# Supplementary material for: Fractional Charge States in the Magneto-Photoluminescence Spectra of Single-Electron InP/GaInP2 Quantum Dots
Source: Nanomaterials (Basel). 2021 Feb 16;11(2):493. doi: 10.3390/nano11020493 (PMC7920047; doi:10.3390/nano11020493)
Supplement: Supplementary file 1 [file nanomaterials-11-00493-s001.pdf]

s1. Trion versus exciton emission in S00 QD.

The PL spectrum of excitonic S00 QD in Fig.SM1a has two components, which can be attributed to a neutral ($S00_{ex}$) and singly charged, trion ($S00_{tr}$) excitonic states. Coexistence of the exciton and the trion in the PL spectrum can indicate a recapture of the hole after the emission of the trion [1]. Exciton and trion components of S00 QD are recognized from their diamagnetic and paramagnetic shift fit to Fock-Darwin (FD) spectrum [2] (see Fig.SM1b), respectively. The diamagnetic coefficient, measured from fitting [3], is 13 μeV/T^2 and corresponds to an exciton radius of ~7 nm, which is close to a Bohr radius a_B^* of InP QDs [4]. The paramagnetic dispersion follows well trion FD spectrum downwards B_e~5 T, where the decomposition of the trion and formation of the 2e WM in PS is expected [2]. For larger fields the paramagnetic dispersion becomes weaker compared to FDL and reveals a small kink at B_e=8 T. These two values of B_e are close to effective Landau level filling factors ν~1 and 1/3, which allows relate them to the signatures indicating a formation of a magneto-electron.

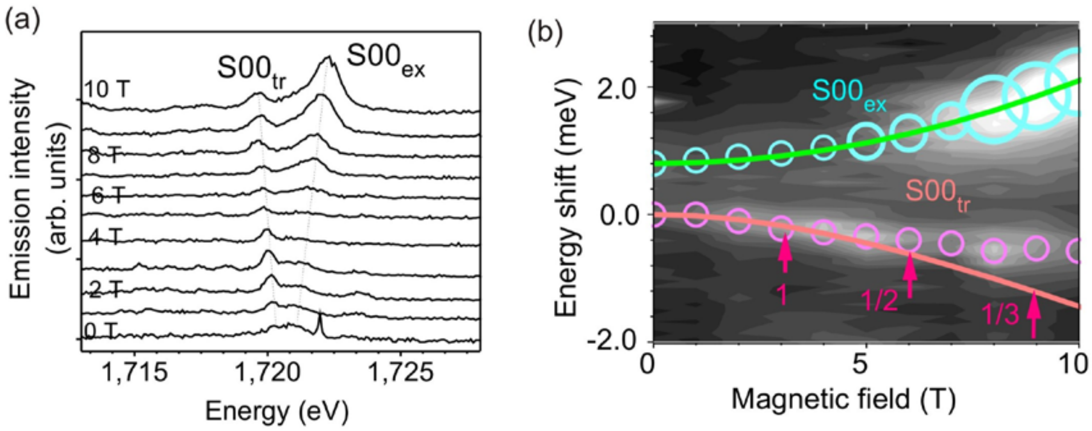

Fig.SM1 (a) Low-temperature (10 K) NSOM PL of S00 $InP/GaInP_2$ QDs measured at B_e=0, 0.5, 1.0, … and 10 T and their contour plot (b), together with calculated FD *B*-dispersion (curves) of exciton ($S00_{ex}$) and trion ($S00_{tr}$). Circles in b are experimental peak position. Dashed curves in (a) outline evolution of peak positions with B_e increase.

[1] M. Ediger, et al. Nature Phys., 3, 774-779 (2007).
[2] A. M. Mintairov, J. Kapaldo, J. L. Merz, A. S. Vlasov and S. A. Blundell, Phys. Rev. B **95**, 115442 (2017)
[3] A. M. Mintairov, et al. Phys. Rev. Lett., **87**, 277401 (2001).
[4] In effective atomic units Bohr radius $a^*_B=\hbar^2(4\pi\varepsilon\varepsilon_0)/m^*e^2$, where ε and m^* are dielectric constant of the material and effective mass of electron, respectively. For our dots it is 8.5 nm.

s2. Electron states of $InP/GaInP_2$ QDs versus size.

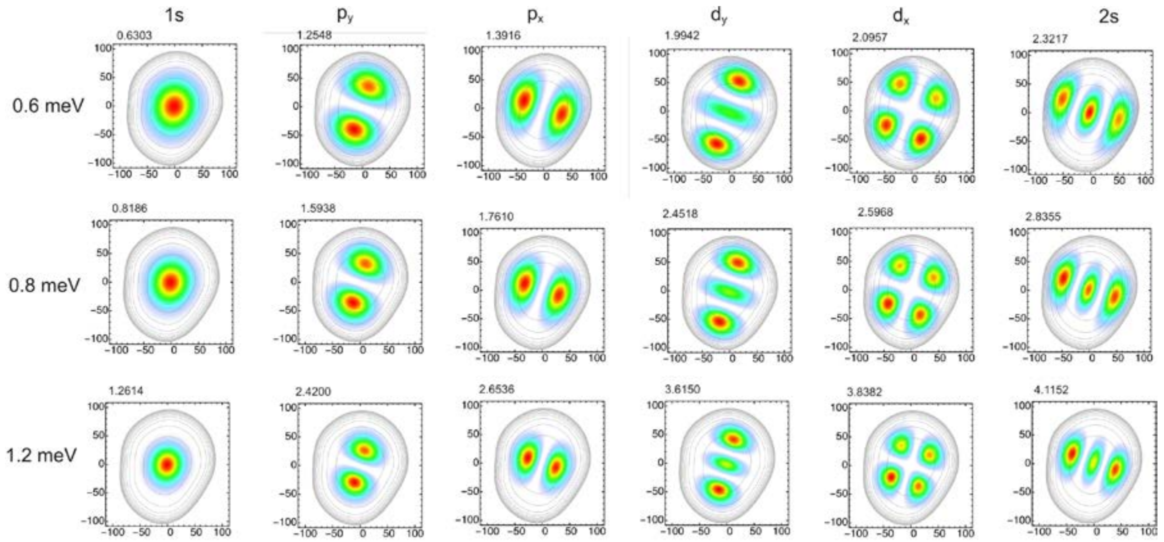

Fig.SM2 Electron density distributions of *s*-, *p*- and *d*-wave single-electron states of $InP/GaInP_2$ QDs having quantum confinement $\hbar\omega_0 = 0.6$, 0.8 and 1.2 meV. Frame size 200x200 nm^2. Numbers above plots are the energy of the level in meV.

s3. Theoretical modeling of electronic structure in magnetic field

We model the quantum dots as a confined 2D electron gas in the effective-mass approximation, with a uniform magnetic field B_z applied perpendicular to the x-y plane of the electrons. The Hamiltonian for N confined electrons is (in atomic units, $4\pi\epsilon_0 = m_e = \hbar = |e| = 1$)

$$H = \sum_{i=1}^{N} \left\{ \frac{1}{2m^*} [\mathbf{p}_i - e\mathbf{A}(\mathbf{r}_i)]^2 + V_{\text{ext}}(\mathbf{r}_i) + g^* \mu_B B_z S_{zi} \right\} + \sum_{i>j}^{N} \frac{e^2}{\epsilon |\mathbf{r}_i - \mathbf{r}_j|} , \qquad \text{(S1)}$$

where $V_{\text{ext}}(\mathbf{r})$ is the effective 2D confining potential in the x-y plane and the magnetic vector potential is given by $\mathbf{A}(x, y) = (B_z/2)(-y, x, 0)$. We take the conduction-band effective mass of InP to be $m^* = 0.077$ and the dielectric constant to be $\epsilon = 12.61$. The appropriate value of the effective g-factor g^* for our quantum dots is unclear to us, so for illustrative purposes the spin Zeeman term is included using the value for bulk InP, $g^* = +1.20$.

The Hamiltonian (S1) is discretized on a 2D Cartesian grid using a high-order (11-point) finite-difference method, adapted to take account of the rapid variation of the phase of the complex wave function between adjacent grid points in the presence of a strong magnetic field. Typical grid dimensions are 70×70 for $B_z < 5$ T (and larger for higher B_z). The many-particle wave functions and energies of confined electrons are computed by a configuration-interaction (CI) approach, which is based on a

generalization of earlier work [1–3] where no magnetic field was present. The first step is to generate a single-particle basis set including states up to a high energy cutoff, using either a spin-polarized Hartree-Fock (HF) potential or an analytical approximation to the HF (mean-field) potential. For $N = 2$ confined electrons, we then find the many-body wave function and total energy by full CI (including up to double excitations) using the HF basis set. To evaluate the Coulomb integrals required, we solve Poisson's equation in the x-y plane by a complex version of the algorithm described in Ref. [1], which employs fast Fourier transforms and a quintic interpolation of the density between grid points. A useful overall check on the many-body calculation is provided by the fact that we can obtain total energies for the $2e$ system that agree to six or more digits with a variety of different basis sets, for the ground state as well as the low-lying excited states.

We model the experimental dots with a simple 2D effective confining potential,

$$V_{\mathrm{ext}}(\mathbf{r}) = \frac{1}{2} R_0^2 \omega_0^2 \left\{ \frac{r^2}{R(\theta)^2} + \exp\left[\alpha\left(r - R(\theta)\right)\right] \right\} . \qquad \text{(S2)}$$

The physical boundary of the dot (where the vertical height of the InP deposit goes to zero) is represented by a radius function $R(\theta)$, which in general depends on the polar angle θ in the x-y plane, so that the dot has an angular deformation. The parameter $R_0 = \langle R(\theta) \rangle$ is the average value of $R(\theta)$ over the polar angle $0 \leq \theta \leq 2\pi$. For small radii $r \ll R(\theta)$, the potential $V_{\mathrm{ext}}(\mathbf{r})$ is approximately harmonic with frequency ω_0, while at large radii $r \gg R(\theta)$, the potential develops a hard wall (controlled by the parameter α), which forces the electron wave function to zero. The angular deformation of $R(\theta)$ is chosen to be typical of the dots synthesized experimentally.

Our grid-based numerical algorithm makes it straightforward to incorporate the symmetry-breaking effect of a deformed dot by direct calculation with the confining potential. As a result of the angular deformation, the orbital angular momentum L_z is not an exact quantum number and localized electron states can be observed directly in the one-body electron density

$$\rho(\mathbf{r}_0) = \sum_{i=1}^{N} \langle \Psi | \delta^2(\mathbf{r}_{\mathrm{i}} - \mathbf{r}_0) | \Psi \rangle . \qquad \text{(S3)}$$

Figure SM3 shows this phenomenon in the ground-state densities for two potentials with harmonic parameters $\hbar\omega_0 = 1.3$ meV (top row) and 0.6 meV (bottom row). The potential contours indicate the deformed shape of the confining potential and its outer physical radius $R(\theta)$; the average physical radius is $R_0 \approx 85$ nm. For dots of this size and shape, even in zero field $B_z = 0$, the ground state is a clearly defined Wigner molecule with two well separated electron peaks. For sufficiently large B_z (in these examples, for $B_z > \sim 2$ T), the peaks become sharper as B_z increases further, reflecting the magnetic length scale $l_B = \sqrt{\hbar/eB}$. For the shallower potential $\hbar\omega_0 =$ 0.6 meV, the peaks are further apart and begin to push up against the hard wall of the

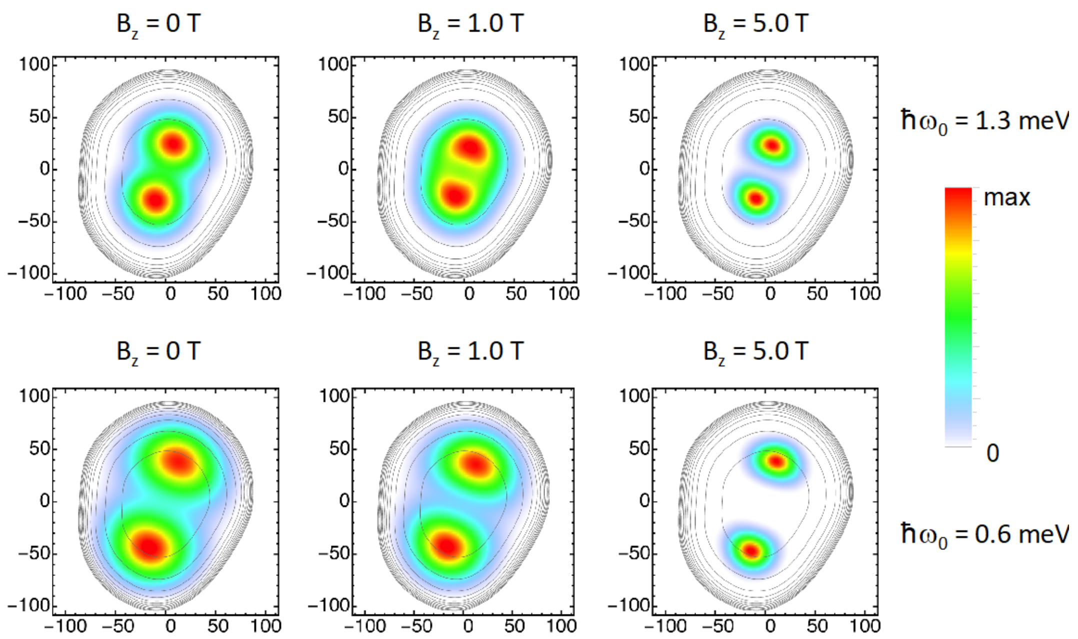

Fig. SM3. Density of $N = 2$ confined electrons (with $S_z = 0$) for harmonic confining frequency $\hbar\omega_0 = 1.3$ meV (top row) and $\hbar\omega_0 = 0.6$ meV (bottom row) and $B_z = 0$ T, 1 T, and 5 T. The contours indicate the deformed confining potential and the x- and y-axes are labeled in nm.

potential, while for $\hbar\omega_0 = 1.3$ meV the confinement is mainly by the central harmonic part of the potential.

In a strong magnetic field, the lowest-energy states with $S_z = 0$ consist of nearly degenerate triplet ($S = 1$) and singlet ($S = 0$) states. The total energy and the splittings of these states as a function of B_z are shown in Figs. SM4a and b for the dot from Fig. SM3 with $\hbar\omega_0 = 0.6$ meV. As B_z increases and the two electrons become more localized, the exchange interaction between them is reduced and the singlet-triplet splitting of the $S_z = 0$ components becomes increasingly small [see Fig. SM4b].

The triplet states with $S_z \neq 0$ are Zeeman-shifted by an energy $g^*\mu_B B_z S_z$ relative to those with $S_z = 0$, so that (assuming $g^* > 0$) the ground state at high B_z is the $S_z = -1$ component of the triplet state. The low-lying excited states in a strong field (not shown in the figure) also have $S_z = -1$ and lie between the curves for $S_z = -1$ and 0 in Fig. SM4(b). These excited states originate from excited triplet states at $B_z = 0$, the $S_z = -1$ components of which are Zeeman-shifted to lower energies as B_z increases.

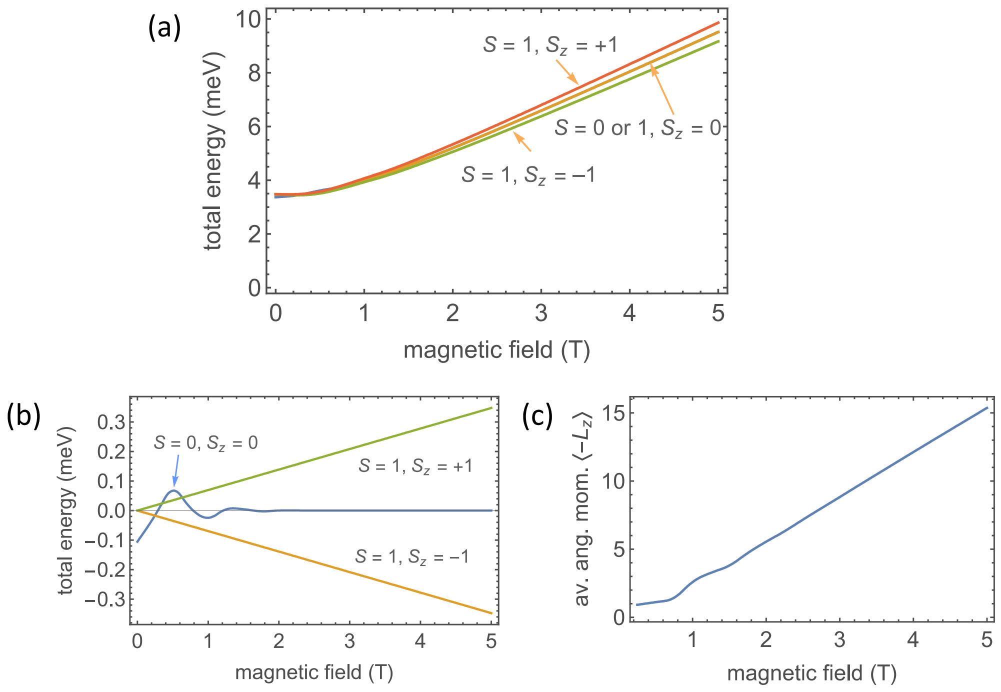

Fig. SM4. (a) Total energy of the ground-state spin multiplets with total spin $S = 1$ and 0; (b) relative total energy of these states, with the zero of energy defined as the energy of the $S = 1, S_z = 0$ state; (c) average orbital angular momentum $\langle L_z \rangle$ of the $S = 1, S_z = -1$ state. The quantum dot is that from Fig. S1 with $\hbar\omega_0 = 0.6$ meV.

For this confining potential, the average orbital angular momentum $\langle L_z \rangle$ of the ground state displays broadened plateaus at values of $\langle L_z \rangle = 1$ and 3 [see Fig. SM4c]. These plateaus are washed out for $\langle L_z \rangle = 7$ and higher, after which $\langle L_z \rangle$ shows a near-linear dependence on B_z.

[1] S. A. Blundell and K. Joshi, Phys. Rev. B **81**, 115323 (2010).
[2] S. A. Blundell and S. Chacko, Phys. Rev. B **81**, 121104(R) (2010).
[3] S. A. Blundell and S. Chacko, Phys. Rev. B **83**, 195444 (2011).
